# Calculation of eigenvalues of Sturm–Liouville equation for simulating hydrodynamic soliton generated by a piston wave maker

**DOI:** 10.1186/s40064-016-2911-0

**Published:** 2016-08-19

**Authors:** A. Laouar, A. Guerziz, A. Boussaha

**Affiliations:** 1 LANOS Laboratory, Department of Mathematics, Badji Mokhtar University of Annaba, P.O. Box 12, 23000 Annaba, Algeria; 2 Department of Physics, Faculty of Sciences, Badji Mokhtar University of Annaba, P.O. Box 12, 23000 Annaba, Algeria

**Keywords:** KdV equation, Soliton-solution, Sturm–Liouville spectral problem, Runge–Kutta algorithm

## Abstract

This paper focuses on the mathematical study of the existence of solitary gravity waves (solitons) and their characteristics (amplitude, velocity, $\ldots$) generated by a piston wave maker lying upstream of a horizontal channel. The mathematical model requires both incompressibility condition, irrotational flow of no viscous fluid and Lagrange coordinates. By using both the inverse scattering method and a given initial potential $f_{0}(r),$ we can transform the KdV equation into Sturm–Liouville spectral problem. The latter problem amounts to find negative discrete eigenvalues $\lambda$ and associated eigenfunctions $\psi$, where each calculated eigenvalue $\lambda$ gives a soliton and the profile of the free surface. For solving this problem, we can use the Runge–Kutta method. For illustration, two examples of the wave maker movement are proposed. The numerical simulations show that the perturbation of wave maker with hyperbolic tangent displacement under physical conditions affect the number of solitons emitted.

## Background

**Fig. 1:**
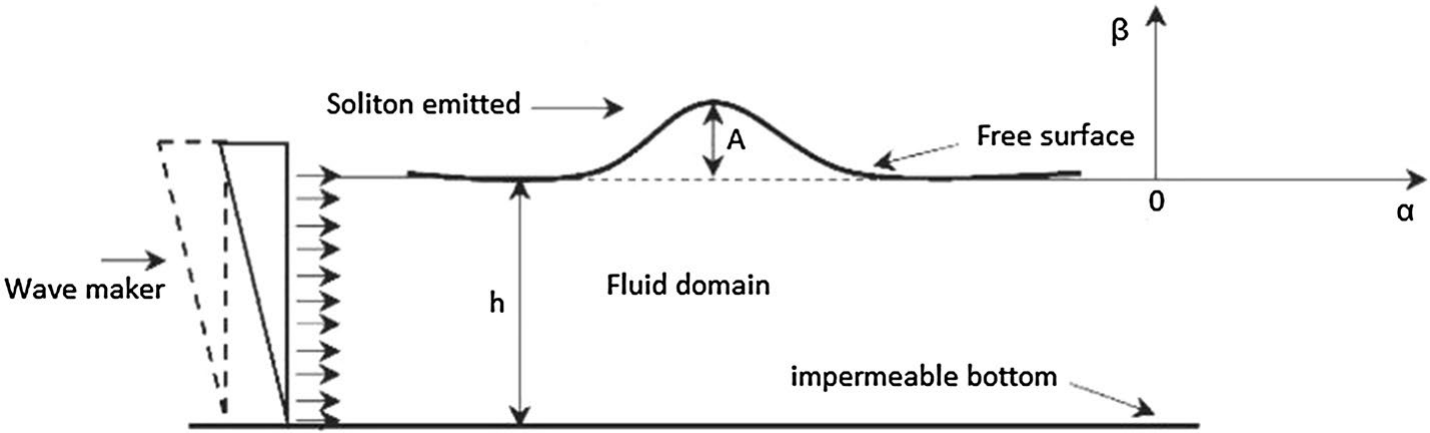
Description of the Phenomenon

Interest in nonlinear wave propagation has grown rapidly during the last three decades and has gained considerable attention in engineering and applied mathematics. This should not be surprising since the nonlinear waves phenomena are presented in many physics areas, such as fluid dynamics, hydrodynamics, optical fibres, plasma physics, biology, etc. As is well known the models describing the phenomenon are often represented by a set of partial differential equations completed by the boundary conditions and initial conditions related to time (see Biswas and Triki 2011; Courant and Hilbert 1953; Germain 1972; Miranville and Temam 2000). For example, the modeling of the phenomena from an hydrodynamic or optical fields can be generally outlined as follows: 
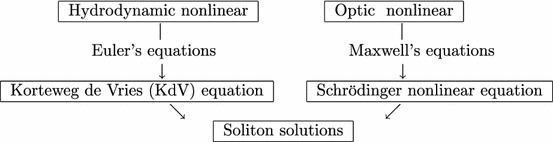


This paper concerns a propagation of surface liquid waves, in general, and the special case linking major solitary gravity waves—called also solitons—which is a topic of interest as well for physicists and mathematicians (see Ablowitz and Clarkson 1991; Biswas and Triki 2011; Germain 1972; Tzirtzilakis et al. 2002). The most model representative in fluid mechanics and best known is based on the Navier–Stokes equations (see Germain 1972; Miranville and Temam 2000). It should be noted that these equations are generally nonlinear phenomenon and the explicit analytical solution is often non-existent; therefore the numerical approach remains the most appropriate approach to treat this phenomenon. Another model represented a swell propagation on horizontal bottom is describing throughout the Boussinesq equations (see Boussinesq 1872; Daripa and Hua 1999) which represent the integration on vertical of both conservation of movement quantity and conservation of mass for an incompressible fluid. These allow considering the transfer of energy between the multiple frequency components and the changing of shape of individual wave and the evolution of a group random waves. The main limitation of the most common form of the Boussinesq equations is that they are only valid for relatively shallow water depths. It was not until the year 1990 that many initial Boussinesq equations derived models have been developed to extend their domains of validity to shallow water and especially by improving the dispersion equation (see Boussinesq 1872; Daripa and Hua 1999; Yao et al. 2007). This work leads to the study of the existence and the physical characteristics of solitary gravity waves (amplitude, speed, $\ldots$). Experimentally, these may be generated by a piston wave maker at the upstream of a horizontal channel (see, Fig. 1). After modeling the phenomenon by a system of equations, it can be transformed, by introducing a double distortion and a fourth order approximation with respect to the parameter of distortion $\varepsilon ,$ into KdV equation (see Gardner et al. 1967; Miranville and Temam 2000; Tzirtzilakis et al. 2002). The latter is given below:

1 $$\begin{aligned} \frac{\partial f}{\partial s}\left( r,s\right) -6f\dfrac{\partial f}{\partial r}\left( r,s\right) +\frac{\partial ^{3}f}{\partial r^{3}}\left( r,s\right) =0, \end{aligned}$$

where *s* and *r* are space and time variables respectively.

The balance between the nonlinear convection term $f\dfrac{\partial f}{\partial r}$ and the dispersion effect term $\frac{\partial ^{3}f}{\partial r^{3}}$ in the spatially one-dimensional KdV equation (1) gives rise to solitons (Gardner et al. 1967). These are defined as localized waves that propagate without change of their shape and velocity properties and stable against mutual collisions (Yao et al. 2007).

The aim of our paper focuses on the study of solitary wave (soliton) generated by piston wave maker placed at upstream. The mathematical model requires both incompressibility condition, irrotational flow of no viscous fluid and Lagrange coordinates. The use of both the inverse scattering method (see Alquran and Al-Khaled 2010; Ablowitz and Clarkson 1991; Aktosun 2005) and a given initial potential $f_{0}(r)$ allow to transform the KdV equation into Sturm–Liouville spectral problem (see Temperville 1985): find the eigenvalues $\lambda$ and associated eigenfunctions $\psi$ such that

2 $$\begin{aligned} \dfrac{d^{2}\psi \left( r\right) }{dr^{2}}+\left( \lambda -f_{0}(r)\right) \psi \left( r\right) =0. \end{aligned}$$

More particularly, the problem amounts to find a negative discrete eigenvalues $\lambda$ and associated eigenfunctions $\psi$, where each calculated eigenvalue $\lambda$ gives a soliton and the profile of the free surface. For solving the problem (2), we can use a numerical method and for illustration, two examples of the wave maker movement are proposed.

The plan of this paper is as follows. Section “Position of the problem” gives the “Description of the phenomenon” section and “Basic equations of the mathematical model” section. Section “Techniques of resolution” comprises three subsections: the introducing of “The distortion variables” section, the approximating solutions with respect to the parameter of distortion $\varepsilon$ and the solution of Sturm–Liouville spectral equation (2) by the Runge–Kutta method. The last section presents numerical applications for illustrating the theoretical model.

## Position of the problem

### Description of the phenomenon

We consider a fixed *Oxy* reference system, where the *y*-axis is vertically ascendant and the *x*-axis coincides with the initial free surface. The position of the fluid particle at the moment *t*, $t>0$, is denoted by (*x*, *y*) and their coordinates at the initial position by (*a*, *b*),  where *a*, *b* and *t* are the Lagrangian variables.

The domain $\Omega =\left\{ x\ge 0\text { and }-h\le y\le 0\right\}$ is occupied by fluid of an infinite horizontal band which is limited vertically by a free surface $b=0$ and an impermeable horizontal bottom $b=-h$. The wave maker type piston placed at upstream $(a=0)$ generates same waves (see, Fig. 1). The new coordinates *X* and *Y* are introduced as follows:

$$\begin{aligned} X(a,b,t)=x(a,b,t)-a\quad \hbox { and }\quad Y(a,b,t)=y(a,b,t)-b. \end{aligned}$$

**Fig. 2:**
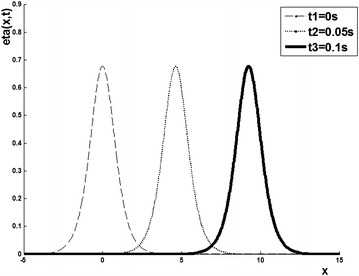
1-Soliton solution

**Fig. 3:**
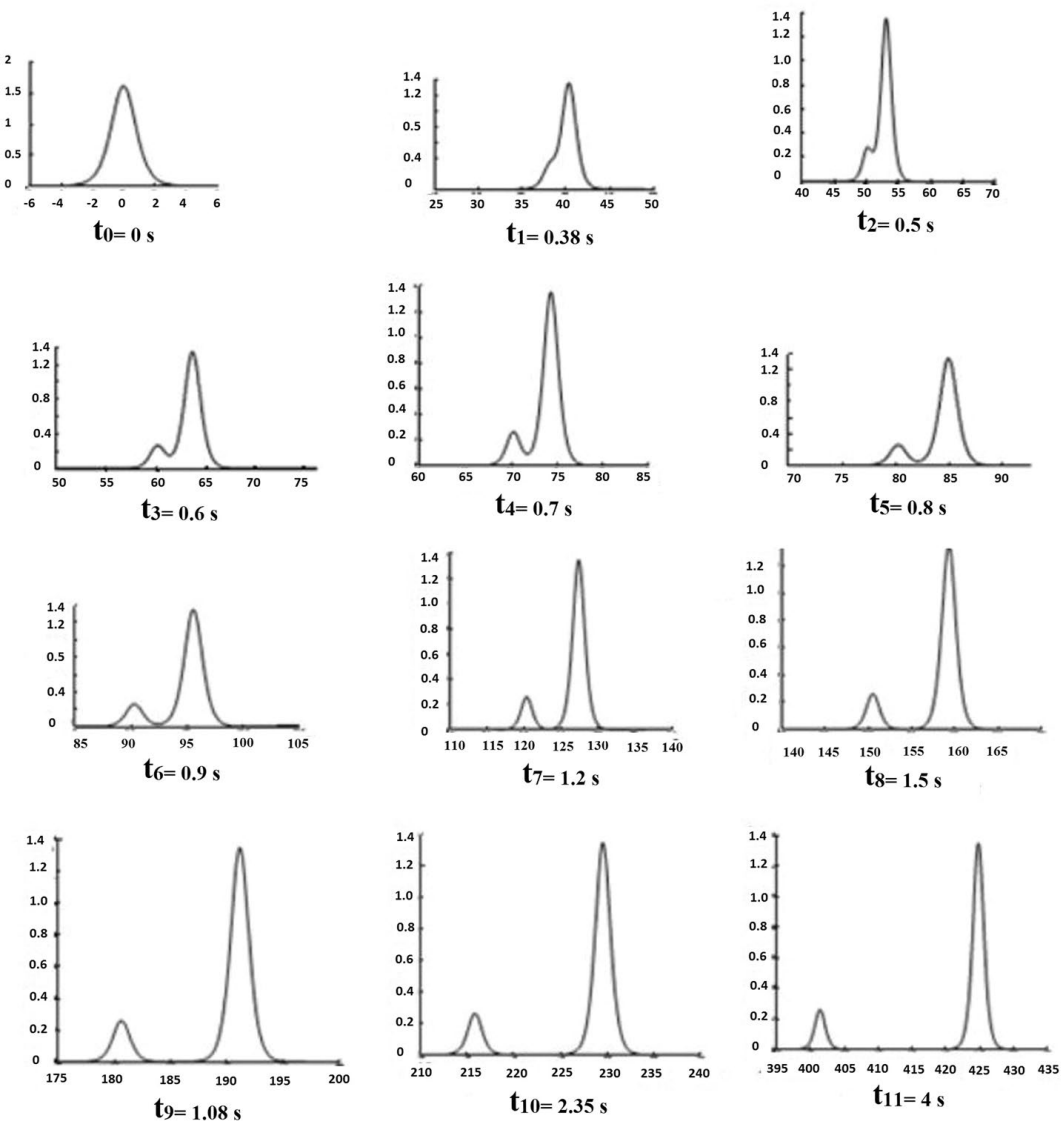
2-Soliton solution: interaction of two solitons

### Basic equations of the mathematical model

General equations and mathematical model are listed below:

(*i*) the kinematic condition expresses the incompressibility of fluid (the Jacobian equals unity)

3 $$\begin{aligned} \frac{\partial X}{\partial a}+\frac{\partial Y}{\partial b}+\frac{\partial X}{\partial \alpha }+\frac{\partial X}{\partial a}\frac{\partial Y}{\partial b}-\frac{\partial X}{\partial b}\frac{\partial Y}{\partial a}=0, \end{aligned}$$

(*ii*) the dynamic condition for an irrotational movement

4 $$\begin{aligned} \frac{\partial ^{2}X}{\partial b\partial t}\left( 1+\frac{\partial X}{\partial a}\right) -\frac{\partial X}{\partial b}\frac{\partial ^{2}X}{\partial a\partial t}+\frac{\partial Y}{\partial a}\frac{\partial ^{2}Y}{\partial b\partial t}-\frac{\partial ^{2}Y}{\partial a\partial t}\left( 1+\frac{\partial Y}{\partial b}\right) =0, \end{aligned}$$

(*iii*) the impermeability boundary conditions

5 $$\begin{aligned}&\left( 1+\frac{\partial X}{\partial a}\right) \frac{\partial ^{2}X}{\partial t^{2}}+\frac{\partial Y}{\partial a}\frac{\partial ^{2}Y}{\partial t^{2}}-g\frac{\partial Y}{\partial a}=0\text { at the free surface } (b=0), \end{aligned}$$

6 $$\begin{aligned}&Y(a,b=-h,t)=0\quad \text {at the bottom}, \end{aligned}$$

(*iv*) the initial conditions

7 $$\begin{aligned} X(a,b,-\infty )=0\quad\text {and}\quad Y(a,b,-\infty )=0\quad \text {at rest }, \end{aligned}$$

(*v*) the piston wave maker equation

8 $$\begin{aligned} X\left( a=0,b,t\right) =D\left( t\right) , \end{aligned}$$

where *D* is a given positive function which represents the elongation of wave maker.

## Techniques of resolution

### The distortion variables

In this part, we transform the Eqs. (3)–(8) into KdV equation (1), for this we introduce distortion variables which express the assumption of shallow water and asymptotic profile of wave respectively. Afterwards we use the approximate solution at fourth order and the inverse scattering method (for more details, see Aktosun 2005) in order to obtain Sturm–Liouville spectral problem.

(*a*) *Classical distortion variables*: the assumption of the shallow water theory (see Germain 1972; Laouar 2008) inserts distortion variables space and temporal, translating the difference in scale between the sizes horizontal and vertical. This distortion will be characterized by using a small parameter $\varepsilon$ as follows:

$$\begin{aligned} \alpha =\varepsilon a,\quad \beta =b\quad \hbox { and }\quad \tau =\varepsilon \sqrt{gh} t, \end{aligned}$$

where $\sqrt{gh}$ represents the critical celerity of the propagated long waves, *h* and *g* are the depth of fluid at rest and the gravity respectively.

The Eqs. (3)–(8) become respectively

9 $$\begin{aligned}&\frac{\partial Y}{\partial \beta }+\varepsilon \left[ \frac{\partial X}{\partial \alpha }+\frac{\partial X}{\partial \alpha }\frac{\partial Y}{\partial \beta }-\frac{\partial X}{\partial \beta }\frac{\partial Y}{\partial \alpha }\right] =0, \end{aligned}$$

10 $$\begin{aligned}&\frac{\partial ^{2}X}{\partial \beta \partial \tau }+\varepsilon \left[ \frac{\partial X}{\partial \alpha }\frac{\partial ^{2}X}{\partial \beta \partial \tau }-\frac{\partial X}{\partial \beta }\frac{\partial ^{2}X}{\partial \alpha \partial \tau }+\frac{\partial Y}{\partial \alpha }\frac{\partial ^{2}Y}{\partial \beta \partial \tau }-\left( 1+\frac{\partial Y}{\partial \beta }\right) \frac{\partial ^{2}Y}{\partial \alpha \partial \tau }\right] =0, \end{aligned}$$

11 $$\begin{aligned}&\frac{\partial Y}{\partial \alpha }+\varepsilon h\frac{\partial ^{2}X}{\partial \tau ^{2}}+\varepsilon ^{2}h\left[ \frac{\partial X}{\partial \alpha }\frac{\partial ^{2}X}{\partial \tau ^{2}}+\frac{\partial Y}{\partial \alpha }\frac{\partial ^{2}Y}{\partial \tau ^{2}}\right] =0\text { at the free surface } (\beta =0), \end{aligned}$$

12 $$\begin{aligned}&Y(\alpha ,\beta ,\tau )=0\quad \text {at the bottom } (\beta =-h), \end{aligned}$$

13 $$\begin{aligned}&X(\alpha ,\beta ,-\infty )=0\text { and }Y(\alpha ,\beta ,-\infty )=0\quad \text {(at rest )}, \end{aligned}$$

14 $$\begin{aligned}&X\left( \alpha =0,\beta ,t\right) =D\left( t\right) . \end{aligned}$$

(*b*) *Double distortion variables*: before reaching their asymptotic profile, the waves will under go from the initial potential a slow evolution. The double distortion is introduced as follows:

15 $$\begin{aligned} \theta =\varepsilon \left( \alpha -\sqrt{gh}t\right) ,\quad \varphi =\varepsilon ^{3}\alpha , \end{aligned}$$

where $\theta$ and $\varphi$ represent the fast and slow variable respectively.

The derivatives with respect to $\theta$ and $\varphi$ are

16 $$\begin{aligned} \frac{\partial }{\partial \alpha }=\varepsilon \frac{\partial }{\partial \theta }+\varepsilon ^{3}\frac{\partial }{\partial \varphi }\quad \text {and}\quad \frac{\partial }{\partial t}=-\varepsilon \sqrt{gh}\frac{\partial }{\partial \theta }. \end{aligned}$$

Using new distortion variables in Eqs. (9)–(13) we obtain respectively

17 $$\begin{aligned}&\dfrac{\partial Y}{\partial \beta }+\varepsilon \left[ \dfrac{\partial X}{\partial \theta }+\dfrac{\partial X}{\partial \theta }\dfrac{\partial Y}{\partial \beta }-\dfrac{\partial X}{\partial \beta }\dfrac{\partial Y}{\partial \theta }\right] +\varepsilon ^{3}\left[ \dfrac{\partial X}{\partial \varphi }+\dfrac{\partial X}{\partial \varphi }\dfrac{\partial Y}{\partial \beta }-\dfrac{\partial X}{\partial \beta }\dfrac{\partial Y}{\partial \varphi }\right] =0, \end{aligned}$$

18 $$\begin{aligned}&\left. \dfrac{\partial ^{2}X}{\partial \beta \partial \theta }+\varepsilon \left[ \dfrac{\partial X}{\partial \theta }\dfrac{\partial ^{2}X}{\partial \beta \partial \theta }-\dfrac{\partial X}{\partial \beta }\dfrac{\partial ^{2}X}{\partial \theta ^{2}}+\dfrac{\partial Y}{\partial \theta }\dfrac{\partial ^{2}Y}{\partial \beta \partial \theta }-\dfrac{\partial Y}{\partial \beta }\dfrac{\partial ^{2}Y}{\partial \theta ^{2}}-\dfrac{\partial ^{2}Y}{\partial \theta ^{2}}\right] \right. \nonumber \\&\quad\quad \left. +\varepsilon ^{3}\left[ \dfrac{\partial X}{\partial \varphi }\dfrac{\partial ^{2}X}{\partial \beta \partial \theta }-\dfrac{\partial X}{\partial \beta }\dfrac{\partial ^{2}X}{\partial \theta \partial \varphi }+\dfrac{\partial Y}{\partial \varphi }\dfrac{\partial ^{2}Y}{\partial \beta \partial \theta }-\dfrac{\partial Y}{\partial \beta }\dfrac{\partial ^{2}Y}{\partial \theta \partial \varphi }-\dfrac{\partial ^{2}Y}{\partial \theta \partial \varphi }\right] =0,\right. \end{aligned}$$

19 $$\begin{aligned}&\dfrac{1}{h}\left[ \dfrac{\partial Y}{\partial \theta }+\varepsilon ^{2}\dfrac{\partial Y}{\partial \varphi }\right] +\varepsilon \left[ \dfrac{\partial ^{2}X}{\partial \theta ^{2}}+\varepsilon \left( \dfrac{\partial X}{\partial \theta }\dfrac{\partial ^{2}X}{\partial \theta ^{2}}+\dfrac{\partial Y}{\partial \theta }\dfrac{\partial ^{2}Y}{\partial \theta ^{2}}\right) +\varepsilon ^{3}\left( \dfrac{\partial X}{\partial \varphi }\dfrac{\partial ^{2}X}{\partial \theta ^{2}}\right. \right. \nonumber \\&\quad\quad \left. \left. \left. +\dfrac{\partial Y}{\partial \varphi }\dfrac{\partial ^{2}Y}{\partial \theta ^{2}}\right) \right] =0,\right. \end{aligned}$$

20 $$\begin{aligned}&Y\left( \theta ,\varphi ,-h\right) =0\quad\text { and }\quad \beta =-h, \end{aligned}$$

21 $$\begin{aligned}&X\left( \theta ,\varphi ,-\infty \right) =0\quad\text { and }\quad Y\left( \theta ,\varphi ,-\infty \right) =0. \end{aligned}$$

### Approximation of the solutions of the eqs. (17)–(21)

According to the classical theory of shallow water (see Germain 1972; Laouar 2008), the solutions are developable entire series in $\varepsilon$ as follows:

22 $$X(\theta ,\varphi ,\beta )= \mathop {\sum }_{n=0}^{\infty }\varepsilon ^{2n+1}X_{2n+1}(\theta ,\varphi ,\beta ),$$

23 $$Y(\theta ,\varphi ,\beta )= \mathop {\sum }_{n=0}^{\infty }\varepsilon ^{2n}Y_{2n}(\theta ,\varphi ,\beta ).$$

Substituting (22) and (23) in (17)–(19) and approximating at fourth order, we choose, among the various approximations, the following

24 $$\begin{aligned} \frac{h^{2}}{3}\frac{\partial ^{4}X_{1}}{\partial \theta ^{4}}-3\frac{\partial X_{1}}{\partial \theta }\frac{\partial ^{2}X_{1}}{\partial \theta ^{2}}+2\dfrac{\partial ^{2}X_{1}}{\partial \theta \partial \varphi }=0. \end{aligned}$$

Using auxiliary variables *r* and *s*

25 $$\begin{aligned} r=\frac{\theta }{\varepsilon h}=\frac{\alpha -\sqrt{gh}t}{h},\quad s=\frac{\varphi }{6\varepsilon ^{3}h}=\frac{\alpha }{6h}, \end{aligned}$$

the expression (24) becomes

26 $$\begin{aligned} \frac{3}{2}\varepsilon ^{2}\frac{\partial X_{1}}{\partial \theta }=-\frac{3}{2}\varepsilon ^{2}\frac{\eta _{2}}{h}=\frac{-3\eta }{2h}+O\left( \varepsilon ^{4}\right) , \end{aligned}$$

where $\eta$ is a free surface $\left( y=\eta (x,t)\right) .$

If we neglect the $O\left( \varepsilon ^{4}\right)$ (see Temperville 1985), the function *f*(*r*, *s*) can be written in the form

27 $$\begin{aligned} f(r,s)\simeq \frac{3}{2}\varepsilon ^{2}\frac{\partial X_{1}}{\partial \theta }=-\frac{3}{2}\varepsilon ^{2}\frac{\eta _{2}}{h}=\frac{-3\eta }{2h}. \end{aligned}$$

which must satisfy the KdV equation (1).

The free surface equation is:

28 $$\begin{aligned} \eta (x,t)=-\frac{2}{3}hf(r,s). \end{aligned}$$

For $\alpha =s=0,$ the function $f_{0}\left( r\right)$ (where $f_{0}\left( r\right) =$*f*(0, *r*)) equals $\dfrac{\partial X_{1}}{\partial \theta }\left( \alpha =0,t\right) .$ The generator of the movement of long waves follows a given law (see Temperville 1985) whose equation is

$$\begin{aligned} \quad D\left( r\right) =X\left( \alpha =0,\beta ,t\right) =\varepsilon X_{1}\left( \alpha =0,t\right) +O\left( \varepsilon ^{3}\right) . \end{aligned}$$

Note that the term $O\left( \varepsilon ^{3}\right)$ can be neglected in all the sequel.

#### Soliton-solution of the KdV equation

Now in order to show that the KdV equation admits as particular solution a solitary wave, we give the proposition below.

##### **Proposition 1**

*The KdV equation* (1) *admits as particular solution a solitary wave (soliton):*

29 $$\begin{aligned} f\left( r,s\right) =\frac{-\mu }{2\cosh ^{2}\left( \frac{\sqrt{\mu }}{2}\left( r-\mu s\right) \right) }, \end{aligned}$$

*where*$\mu$*is an arbitrary parameter.*

##### *Proof*

Putting

30 $$\begin{aligned} f\left( r,s\right) =\phi \left( \xi \right) =\phi \left( r-\mu s\right) , \end{aligned}$$

where $\xi =r-\mu s$ and $\mu$ is an arbitrary parameter.

Substituting $\phi$ in (1), we obtain the following differential equation:

31 $$\begin{aligned} \left( -\mu -6\phi \right) \frac{d\phi }{d\xi }+\frac{d^{3}\phi }{d\xi ^{3}}=0. \end{aligned}$$

By integration, it becomes

32 $$\begin{aligned} -3\phi ^{2}-\mu \phi +\frac{d^{2}\phi }{d\xi ^{2}}=l_{1}, \end{aligned}$$

where $l_{1}$ is a constant.

Multiply the Eq. (32) by $\dfrac{d\phi }{d\xi }$ and integrate, it comes

33 $$\begin{aligned} -\phi ^{3}-\frac{\mu }{2}\phi ^{2}+\frac{1}{2}\left( \frac{d\phi }{d\xi }\right) ^{2}=l_{1}\phi +l_{2}; \end{aligned}$$

the constants $l_{1}$ and $l_{2}$ are determined by using boundary conditions: the wave is flat at infinity; and therefore $\phi$ and its derivatives vanish at infinity $\xi$; this gives $l_{2}=0.$

The derivative of (33) with respect to $\xi$ and simplification $\left( \hbox {division by } \dfrac{d\phi }{d\xi }\right)$ yield

34 $$\begin{aligned} -3\phi ^{2}-\mu \phi +\frac{d^{2}\phi }{d\xi ^{2}}=l_{1}, \end{aligned}$$

therefore $$l_{1}=0$$ when $$\xi \rightarrow \infty .$$

Substituting $l_{1}=l_{2}=0$ in ( 33) and integrating elementary transcendental functions, we obtain the solution

35 $$\begin{aligned} \phi =\phi \left( \xi \right) =\frac{-\mu }{2\cosh ^{2}\left( \frac{\sqrt{\mu }}{2}\xi \right) }, \end{aligned}$$

then

36 $$\begin{aligned} f\left( r,s\right) =\frac{-\mu }{2\cosh ^{2}\left( \frac{\sqrt{\mu }}{2}\left( r-\mu s\right) \right) }. \end{aligned}$$

It is easy to verify that the function *f* is a solution of the KdV equation (1). Note that *f* is practically zero when $\xi$ is taken some units (e.g. $\sqrt{\mu }\left| \xi \right| =\sqrt{\mu }\left| r-\mu s\right| \ge 20$) (see, Miranville and Temam 2000). $\square$

The solution of the KdV equation (1) corresponding to the reflections potential can be asymptotically represented as a superposition of *N* single-soliton solutions propagating to the right and ordered in space by their speeds. For this, we give the proposition below.

##### **Proposition 2**

*The function* (36) *is asymptotically represented by a linear superposition*

37 $$\begin{aligned} f\sim \sum _{n=1}^{N}f_{n}, \end{aligned}$$

*where*

38 $$\begin{aligned} f_{n}\left( r,s\right) =\dfrac{-2K_{n}^{2}}{\cosh ^{2}[K_{n}(r-4K_{n}^{2}s)+\delta _{n}]},\quad n=\overline{1,N}, \end{aligned}$$

*with*$K_{n}$*a number to calculate and*$\delta _{n}$ is *the phase shift dependent of*$K_{n}$.

##### *Proof*

(cf, Temperville 1985). $$\square $$

### The Sturm–Liouville equation

As we said previously, the solution of the KdV equation can be transformed to the Sturm–Liouville linear ordinary differential equation (for more details, see Alquran and Al-Khaled 2010; Temperville 1985); add to this the boundary conditions, the problem becomes:

For a given potential $$f_{0}(r),$$ find the eigenvalues $$\lambda \in \mathbb {R} $$ and the eigenfunctions $$\psi $$$$(\psi (r)\ne 0,$$ for any $$r\in \mathbb {R} )$$ such that

39 $$\begin{aligned} \left\{ \begin{array}{l} \dfrac{d^{2}\psi \left( r\right) }{dr^{2}}+\left( \lambda -f_{0}(r)\right) \psi \left( r\right) =0, \\ \psi \left( -\infty \right) =\psi \left( +\infty \right) =0.\end{array}\right. \end{aligned}$$

The function $f_{0}(r)$, here, is taken as follows:

40 $$\begin{aligned} f_{0}\left( r\right) =\left\{ \begin{array}{l} \dfrac{3}{2h}\dfrac{dD\left( r\right) }{dr}\quad r\in \left[ r_{1},0\right] , \\ 0\quad \quad \quad\quad r\notin \left[ r_{1},0\right] ,\end{array}\right. \end{aligned}$$

where $$r_{1}=-\sqrt{g/h}$$$$t_{1}$$; $$t_{1}$$ is the time at stops wave maker.

*Direct spectral problem*: for a given potential $f_{0}\left( r\right) ,$ the problem (39) is to find the set $\{\lambda \}$ of the admissible values for $\lambda$ and to construct the corresponding eigenfunctions $\psi (r,\lambda ).$ We assume the satisfied Faddeev’s condition (see, Grimshaw 2007)

41 $$\begin{aligned} \int _{-\infty }^{+\infty }\left( 1+\left| r\right| \right) \left| f_{0}(r)\right| \,dr<\infty . \end{aligned}$$

The upper bound for the number N of solitons-solutions can be estimated by the formula (see, Grimshaw 2007)

42 $$\begin{aligned} N\le 1+\mathop {\int }\limits _{-\infty }^{+\infty }\left| r\right| \left| f_{0}\left( r\right) \right| dr. \end{aligned}$$

The spectrum comprises a continuous and discrete spectrum. Note that the Continuous Spectrum $\left( \lambda >0\right)$, called scattering solutions, is not our objective in this study [for more details, see Grimshaw (2007) and Temperville (1985)]

*Discrete Spectrum *$\left( \lambda =\lambda _{n}<0\right) :$* (bound states)*

If the potential $$f_{0}(r)$$ is sufficiently negative near the origin of the $$x-$$axis, the spectral problem (39) implies existence of finite number (see, pp. 416–418, Sulem 1999) of bound states $$\psi =\psi _{n}\left( r;\lambda \right) ,$$$$n=1, \ldots , N$$ corresponding to the discrete admissible values of the spectral parameter $$\lambda =\lambda _{n}=-K_{n}^{2},$$$$K_{n}\in \mathbb {R} ,$$ where $$K_{1}>K_{2}>\cdots >K_{N}.$$

Each eigenvalue $\lambda _{n}=-K_{n}^{2}$ permits to determine the function $f\left( r,s\right)$ which is a soliton.

$K_{n}$-*Conditions* The solution of “The Sturm–Liouville equation” section is to integrate (39) and take into account the continuous solutions and their derivatives which vanish at infinity (see, Temperville 1985).

To solve (39), three cases are to be considered

$\alpha )$ if $r\in ]-\infty ,r_{1}[$ then $f_{0}(r)=0$, the Eq. (39) becomes

43 $$\begin{aligned} \dfrac{d^{2}\psi _{n}\left( r\right) }{dr^{2}}-K_{n}^{2}\psi _{n}\left( r\right) =0, \end{aligned}$$

whose solution is

44 $$\begin{aligned} \widehat{\psi }_{n}\left( r\right) =e^{K_{n}\left( r-r_{1}\right) }, \end{aligned}$$

which satisfies the boundary conditions imposed when $r\rightarrow r_{1}$

45 $$\begin{aligned} \widehat{\psi }_{n}\left( r_{1}\right) =1\quad \text { and } \quad \frac{d\widehat{\psi }_{n}\left( r_{1}\right) }{dr}=K_{n}. \end{aligned}$$

$\beta )$ if $r\in [r_{1},0]$, the Eq. (39) can be solved by the Runge–Kutta algorithm at fourth order. By using (45), we can calculate

46 $$\begin{aligned} \widehat{\psi }_{n}\left( 0\right) \quad\text { and }\quad\frac{d\widehat{\psi }_{n}\left( 0\right) }{dr}. \end{aligned}$$

$\gamma )$ if $r\in \left] 0,\infty \right[$ then $f_{0}(r)=0,$ the general solutions of (39) obtained by Fourier method is

47 $$\begin{aligned} \widehat{\psi }_{n}\left( r\right) =c_{1}e^{-K_{n}r}+c_{2}e^{K_{n}r}. \end{aligned}$$

The coefficients $$c_{1}$$ and $$c_{2}$$ are calculated by using the continuity conditions of $$\widehat{\psi }_{n}$$ and its the derivative at $$r=0 $$. These yield

48 $$\begin{aligned} c_{1}= & {} \frac{1}{2}\left[ \widehat{\psi }_{n}\left( 0\right) -\frac{1}{K_{n}}\frac{d\widehat{\psi }_{n}}{dr}\left( 0\right) \right] , \end{aligned}$$

49 $$\begin{aligned} c_{2}= & {} \frac{1}{2}\left[ \widehat{\psi }_{n}\left( 0\right) +\frac{1}{K_{n}}\frac{d\widehat{\psi }_{n}}{dr}\left( 0\right) \right] . \end{aligned}$$

The bounded solution is obtained if the coefficient $c_{2}$ tends to zero, when $r\rightarrow +\infty$, then $K_{n}$ must verify the following relation

50 $$\begin{aligned} K_{n}\widehat{\psi }_{n}\left( 0\right) +\frac{d\widehat{\psi }_{n}}{dr}\left( 0\right) =0, \end{aligned}$$

the solution of this equation can be obtained by integration. Note that the Eq. (50) is very interesting numerically since it permits to obtain the discrete number $K_{n}$ which is calculated by using the sweeping method in the interval $\left[ 0,K_{\max }\right]$. The number $K_{\max }$ can be obtained throughout the proposition below.

#### **Proposition 3**

*Let the solution* (44) *and the condition* (45), *then we have*

51 $$\begin{aligned} K_{\max }=\sqrt{\sup \left| f_{0}\left( r\right) \right| }. \end{aligned}$$

#### *Proof*

Suppose that

52 $$\begin{aligned} K_{n}>\sqrt{\sup \left| f_{0}\left( r\right) \right| }, \end{aligned}$$

this entrains that

53 $$\begin{aligned} K_{n}^{2}+f_{0}\left( r\right) >0,\quad\forall r. \end{aligned}$$

According to (44), we have:

54 $$\begin{aligned} \widehat{\psi }_{n}\left( r_{1}\right) =1\text { and }\frac{d\widehat{\psi }_{n}\left( r_{1}\right) }{dr}=K_{n}>0, \end{aligned}$$

therefore

$$\begin{aligned} \frac{d^{2}\widehat{\psi }}{dr^{2}}\left( r_{1}\right) >0. \end{aligned}$$

We deduce that $\widehat{\psi }_{n}$ and $\frac{d\widehat{\psi }_{n}}{dr}(r)$ are increasing positive functions. The function $\psi ,$ its first derivative $\frac{d\psi }{dr}$ and second derivative $\frac{d^{2}\psi }{dr^{2}}$ grow with positive eigenvalues, then the relation (50) is not verified; so the (52) is false. $\square$

### Free surface equations

The free surface equation can be written as follows:

55 $$\begin{aligned} \eta \left( x,t\right) =\sum _{n=1}^{N}\frac{A_{n}}{\cosh ^{2}\left( \phi _{n}\right) },\quad\text { for } n=\overline{1,N} \end{aligned}$$

where

56 $$\begin{aligned} \phi _{n}=\frac{xK_{n}}{h}\left( 1-\frac{2}{3}K_{n}^{2}\right) -\sqrt{\dfrac{g}{h}}K_{n}t+\delta _{n}, \end{aligned}$$

with

57 $$\begin{aligned} A_{n}=\frac{4}{3}hK_{n}^{2}, \end{aligned}$$

$A_{n}$ is an amplitude of the *n*-the soliton (Temperville 1985).

If we neglect $\delta _{n}$ and know the $K_{n}$, the free surface equation becomes

58 $$\begin{aligned} \eta \left( x,t\right) =\sum _{n=1}^{N}\frac{A_{n}}{\cosh ^{2}\left( x-c_{n}t\right) },\quad\text { for }n=\overline{1,N} \end{aligned}$$

where

59 $$\begin{aligned} c_{n}=\sqrt{gh}\left( 1+\frac{A_{n}}{2h}\right) , \end{aligned}$$

$c_{n}$ is the velocity of the *n*-the soliton.

## Numerical applications

We have to solve numerically the problem by using Runge–Kutta and Heun methods

60 $$\begin{aligned} \left\{ \begin{array}{l} \dfrac{d^{2}\psi _{n}\left( r\right) }{dr^{2}}-\left( K_{n}^{2}+f_{0}(r)\right) \psi _{n}\left( r\right) =0, \quad \text { for all }r\in \left[ r_{1},0\right] \\ \widehat{\psi }_{n}\left( r_{1}\right) =1\text { and }\frac{d\widehat{\psi }_{n}\left( r_{1}\right) }{dr}=K_{n}>0,\end{array}\right. \end{aligned}$$

We rewrite (60) as a system of first order equations. Putting

$$\begin{aligned} u=\psi \quad \hbox { and } \quad v=\dfrac{du}{dr}, \end{aligned}$$

the (60) can be written as follows

61 $$\begin{aligned} \left\{ \begin{array}{l} v\left( r\right) =\dfrac{du}{dr}\left( r\right) , \\ \dfrac{dv}{dr}\left( r\right) =\left( K^{2}+f_{0}(r)\right) u\left( r\right) ,\quad\text { for all }r\in \left[ r_{1},0\right] , \\ u\left( r_{1}\right) =1,\quad v\left( r_{1}\right) =K_{n}.\end{array}\right. \end{aligned}$$

At *M* equally spaced numbers in the interval $\left[ r_{1},0\right]$ and $K_{n}$ is fixed when *N* equally spaced numbers in the interval $\left[ 0,K\max \right]$.

INPUT: the time at stops wave maker $t_{1},$ elongation *e*, depth of fluid at rest *h*, gravity *g*, tolerance *TOL*,  end point $r_{1}$, integers *N* and *M*, and

$$\begin{aligned} K_{\max }=\dfrac{3\pi e}{2t_{1}\sqrt{gh}}. \end{aligned}$$

1*stapplication*$$N=1,2.$$

The equation of displacement of the piston wave maker follows the given theoretical low:

62 $$\begin{aligned} D\left( r\right) =\left\{ \begin{array}{l} e\left[ 1-\cos \left( \omega r\sqrt{\dfrac{h}{g}}\right) \right] \qquad \text {all }r\in \left[ r_{1},0\right] , \\ 0\qquad r\notin \left[ r_{1},0\right] ,\end{array}\right. \end{aligned}$$

where $$\omega $$$$\left( \omega =\dfrac{\pi }{t_{1}}\right) $$ is the pulsation.

According to (40), the function $f_{0}$ is taken

63 $$\begin{aligned} f_{0}\left( r\right) =\dfrac{3\pi e}{2t_{1}\sqrt{gh}}\sin \left( \dfrac{\pi r}{t_{1}}\sqrt{\frac{h}{g}}\right) ,\quad r\in \left[ r_{1},0\right] . \end{aligned}$$

We take the following data (Table 1)

**Table 1:** The time at stops wave maker $t_{1}$, elongation *e*, depth of fluid *h*, integer *N*, gravity *g* and tolerance for sinusoidal movement displacement of the piston wave maker

$t_{1}$ (s)	*e* (cm)	*h* (cm)	*N*	$\bigtriangleup K_{n}$	$\varepsilon$	*g* $\left( \hbox {cm}/\hbox {s}^{2}\right)$
1.5	10	8	$10^{3}$	$10^{-4}$	$6\times 10^{-7}$	981

We obtain one $K_{n}\,(n=1$) which implies the existence of one soliton

**Table:** 

$K\max$	$K_{n}$	$A (\hbox {cm})$	$c (\hbox {cm}/\hbox {s})$
0.5955	0.2148	0.6152	0.6152

The free surface of one soliton at times $$t_{1}=0$$$$s,~t_{2}=0.05$$*s* and $$t_{3}=0.1$$*s*,  is given by the graph (see, Fig. 2).

Now, we take the following data (Table 2)

**Table 2:** New data: The time at stops wave maker $t_{1}$, elongation *e*, depth of fluid *h*, integer *N*, gravity *g* and tolerance for sinusoidal movement displacement of the piston wave maker

$t_{1}(s)$	*e*( cm)	*h*(cm)	*N*	$\bigtriangleup K_{n}$	$\varepsilon$	$g\left( \hbox {cm}/\hbox {s}^{2}\right)$
1.8	11	10	$5\times 10^{5}$	$2\times 10^{-3}$	$4\times 10^{-7}$	981

The Table 2 gives $K_{n}$ ($n=1,2$) which implies the existence of two solitons (see Fig. 3)

**Table:** 

$K\max$	$K_{1}$	$K_{2}$	$A_{1}$(cm)	$c_{1}(\hbox {cm}/\hbox {s})$	$A_{2}$(cm)	$c_{2}(\hbox {cm}/\hbox {s})$
0.5392	0.318	0.14	1.3483	106.2054	0.2613	100.3568

*Comment*: this example shows the propagation of two solitons: the soliton of high amplitude (1.35 cm associated with the eigenvalue $K1=0.32$) and small amplitude soliton (0.26 cm associated with the eigenvalue $K2=0.14$) and their transient collision. A soliton propagates more quickly than its amplitude is large and if two solitons of different amplitudes are created, there is a collision that does not change the shape of the waves.

2*nd application*$$N=2,3$$

The wave maker $D\left( r\right)$ follows a theoretical law of the motion that generates almost solitons in the absence of “tail” [5].

64 $$D\left( r\right)= e\left[ 1+\tanh \left( -2.48r\sqrt{\frac{h}{g}}-3\right) \right],$$

65 $$f_{0}\left( r\right)= \dfrac{-3.72e}{\sqrt{gh}}\left[ 1-\tanh ^{2}\left( -2.48r\sqrt{\frac{h}{g}}-3\right) \right] , \quad r\in \left[ r_{1},0\right].$$

We take the following data (Table 3)

**Table 3:** The time at stops wave maker $t_{1}$, elongation *e*, depth of fluid *h*, integer *N*, gravity *g* and tolerance for hyperbolic tangent movement displacement of the piston wave maker in the absence of “tail”

$t_{1}(s)$	*e*(cm)	*h*(cm)	*N*	$\bigtriangleup K_{n}$	$\varepsilon$	$g(\hbox {cm}/\hbox {s}^{2})$
2	11	10	$10^{3}$	$10^{-3}$	$10^{-5}$	981

**Fig. 4:**
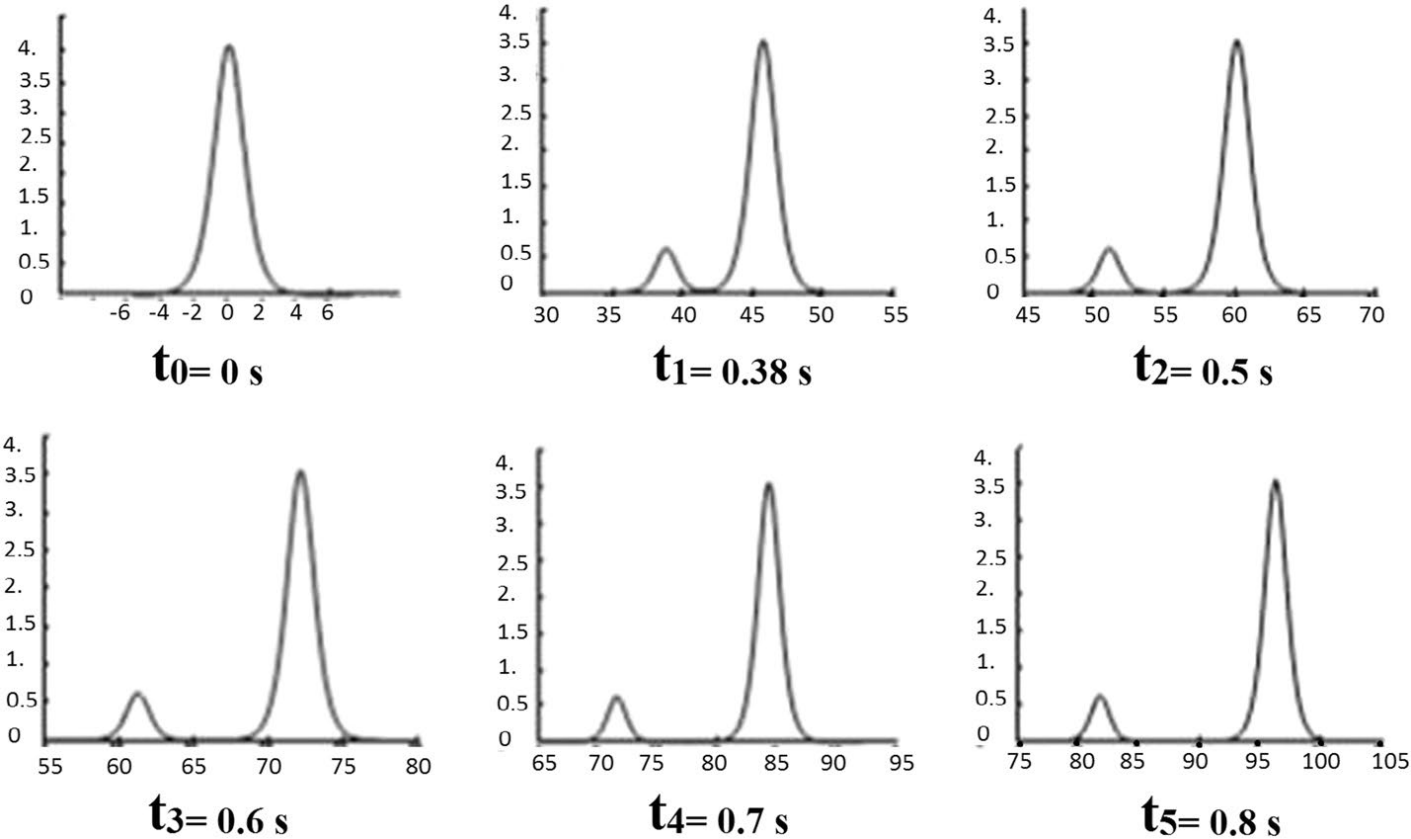
2-Soliton solution in absence of ‘tail’

We give the results of two solitons (see Fig. 4)

**Table:** 

$K\max$	$K_{1}$	$K_{2}$	$A_{1}(\hbox{cm})$	$c_{1}(\hbox{cm}/s)$	$A_{2}(\hbox{cm})$	$c_{2}(\hbox{cm}/s)$
0.6428	0.516	0.216	3.5501	120.4206	0.6221	102.2251

Now, we modify the data as follows (Table 4):

**Table 4:** The time at stops wave maker $t_{1}$, elongation *e*, depth of fluid *h*, integer *N*, gravity *g* and tolerance for hyperbolic tangent movement displacement of the piston wave maker

$t_{1}(s)$	*e*(cm)	*h*(cm)	*N*	$\bigtriangleup K_{n}$	$\varepsilon$	$g(\hbox {cm}/\hbox {s}^{2})$
2	10.3	10	10$^{3}$	10$^{-3}$	10$^{-5}$	981

**Fig. 5:**
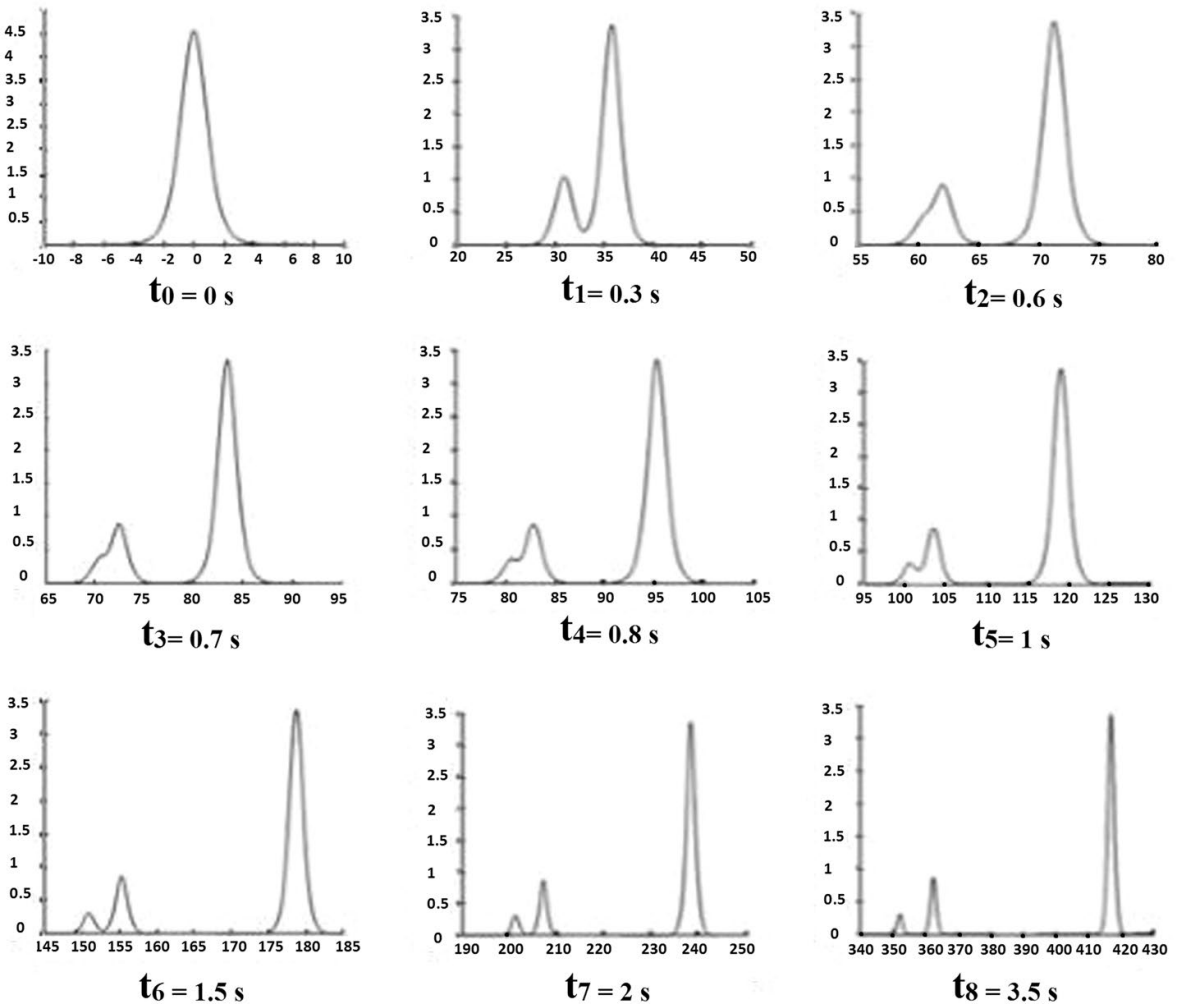
3-Soliton solution: interaction of three solitons

We obtain the results below (see Fig. 5)

**Table:** 

$K\max$	$K_{1}$	$K_{2}$	$K_{3}$	$A_{1}(\hbox{cm})$	$c_{1}(\hbox{cm}/s)$	$A_{2}(\hbox{cm})$	$c_{2}(\hbox{cm}/s)$	$A_{3}(\hbox{cm})$	$c_{3}(\hbox{cm}/s)$
0.6220	0.503	0.256	0.154	3.3735	119.1413	0.8738	103.5705	0.3162	100.6366

### *Remark 4*

To check the validity of our results, it is insightful to compare the obtained soliton solutions with localized pulses propagating in other nonlinear media such as optical waveguides. In this setting, the dynamics of optical solitons is governed by the well-known nonlinear Schrödinger (NLS) equation which is completely integrable by the inverse scattering transform (Ablowitz and Segur 1981). To search for various soliton solutions for the NLS family of equations, many powerful numerical and analytical methods have been recently established and developed. For instance, the finite element method (Kaisar 2008), the ansatz scheme (see, Xu et al. 2016; Zhou et al. 2014), the coupled amplitude-phase formulation (Du et al. 1995; Palacios et al. 1999), variable parametric method (Zhang and Yi 2008), Darboux-Bäcklun transform, and the inverse scattering transform (Zhou et al. 2014) have been successfully applied to exactly solve these models. If one compares the solitary wave profile of the KdV equation presented in Fig. 2 with the bright soliton profile of the NLS equation reported in Du et al. (1995), we can see that there is a certain resemblance. The only noticeable difference is the functional form of the soliton solution in these two models. In fact, the solitary wave solution of the KdV equation is given in terms of “sech2” function (Eq. 36) which differs from the one with “sech” profile for the NLS equation [see Eq. (18) in Du et al. (1995)].

## Conclusion

 This work is devoted to the generation of KdV type solitary wave, obtained by the initial potential $f_{0}(r)$. We have considered two types of movement, either sinusoidal or hyperbolic tangent. The obtained results show that one can therefore control the number of solitons generated by judicious choice of potential $f_{0}(r)$ and physical parameters: positive elongation *e*, depth *h* and time *t* of the displacement. These results will be further expanded in the future. Our next goal is to study the influence of an irregular bottom (*h* depends on the variable *x*) or the presence of an isolated obstacle on the propagation of the solitary wave.
